# Associations of obesity with chronic inflammatory airway diseases and mortality in adults: a population-based investigation

**DOI:** 10.1186/s12889-024-18782-6

**Published:** 2024-05-13

**Authors:** Shanshan Liu, Hao Zhang, Zhihui Lan

**Affiliations:** 1 Jiangxi University of Chinese Medicine, No. 1688, Meiling Avenue, Xinjian District, Nanchang City, Jiangxi Province, China; 2 Department of Cardiology, Donghu District, Affiliated Hospital of Jiangxi University of Traditional Chinese Medicine, No. 445, Bayi Avenue, Nanchang City, Jiangxi Province, China; 3 Department of Respiratory and Critical Care, Donghu District, Affiliated Hospital of Jiangxi University of Traditional Chinese Medicine, No. 445, Bayi Avenue, Nanchang City, Jiangxi Province, China

**Keywords:** Obesity, Chronic inflammatory airway diseases, COPD, Asthma, Chronic bronchitis, NHANES, Mortality

## Abstract

**Background:**

The association between obesity and respiratory diseases has been confirmed. However, few studies have reported the relationship between obesity and the risk and mortality of chronic inflammatory airway disease (CIAD). The aim of this study was to reveal the association between obesity and the risk of CIAD, and mortality in patients with CIAD.

**Methods:**

The study was conducted using data from the National Health and Nutrition Examination Survey (NHANES) 2013 to 2018 among adults aged 20 years and above. All participants were grouped according to body mass index (BMI) and waist circumference (WC) levels to study the relationship between obesity and CIAD. Multivariate logistic regression analysis was utilized to examine the connection between CIAD and obesity in a cross-sectional study. The association between obesity and all-cause mortality in individuals with CIAD was examined using multiple cox regression models and smooth curve fitting in a prospective cohort study.

**Results:**

When stratified based on BMI in comparison to the normal weight group, the ORs with 95%CIs of CIAD for underweight and obesity were 1.39 (1.01–1.93) and 1.42 (1.27–1.58), respectively. The OR with 95%CI of CIAD for obesity was 1.20 (1.09–1.31) when stratified according to WC. Additionally, underweight was associated with a higher mortality (HR = 2.44, 95% CI = 1.31–4.55), whereas overweight (HR = 0.58,95% CI = 0.39–0.87) and obesity (HR = 0.59,95% CI = 0.4–0.87) were associated with a lower mortality (*P* for trend < 0.05). There was a non-linear association between BMI and all-cause mortality (P for non-linear = 0.001). An analysis of a segmentation regression model between BMI and all-cause mortality revealed a BMI turning point value of 32.4 kg/m^2^. The mortality of CIAD patients was lowest when BMI was 32.4 kg/m^2^. When BMI ≤ 32.4 kg/m^2^, BMI was inversely associated with all-cause mortality in patients with CIAD (HR: 0.92, 95%CI:0.88–0.97). However, when BMI > 32.4 kg/m^2^, there was no association between BMI and all-cause mortality (HR:1.02, 95%CI:0.97–1.06).

**Conclusion:**

Compared to normal weight, underweight and obesity were associated with the increased risk of CIAD. Underweight was associated with increased all-cause mortality, while overweight was associated with reduced all-cause mortality. There was a non-linear association between BMI and all-cause mortality in patients with CIAD. The all-cause mortality was lowest when BMI was 32.4 kg/m^2^.

## Introduction

Chronic obstructive pulmonary disease (COPD), asthma and chronic bronchitis are collectively referred to as chronic inflammatory airway diseases(CIAD) and have become an important cause of the increasing global disease burden [1, 2]. According to the Global Burden of Disease 2017 data reported, smoking, household air pollution from solid fuels and ambient particulate matter were the leading risk factors for chronic respiratory disease-related disability [2]. In 2017, an estimated 3.91 million fatalities were attributed to chronic respiratory diseases, marking an increase of 18% compared to 1990 data. Chronic respiratory diseases have become the third leading cause of mortality globally, following cardiovascular diseases and cancer [3]. COPD and asthma are two primary types of chronic respiratory diseases. Both conditions are intricately associated with chronic airway inflammation. In 2019, 212.3 million people worldwide had COPD, resulting in 3.3million deaths and 74.4 million disability adjusted life years [4]. Furthermore, asthma represents a diverse clinical syndrome impacting over 300 million individuals globally, with 25 million affected in the United States alone [5].

Obesity is a pressing global public health concern, and the persistently rising burden is expected to intensify even further [6]. In 2017, there were 4 million people died as a result of being overweight or obese according to global burden of disease statistics [7]. An investigation shown that a total of 124,076 deaths were attributed to obesity-related causes from 1999 to 2020 in the United States [8]. It is well known that obesity is a risk factor for many chronic diseases. Studies have confirmed that obesity affects lung function and outcomes in COPD patients. Another study proposed that body mass index(BMI) and abdominal obesity were associated with the risk of airflow obstruction [9]. Specifically speaking, abdominal obesity increases risk of airway obstruction compared with normal weight population. Additionally, overweight are associated with increased risk of asthma attacks, and population with obesity are at increased risk of asthma exacerbations and hospitalizations [10]. However, despite numerous studies on the relationship between obesity and respiratory diseases, there is no consistent conclusion. Moreover, few studies have reported the relationship between obesity and the risk and mortality of CIAD. Thus we analyzed the data from the National Health and Nutrition Examination Survey (NHANES) 2013–2018 to contribute more scientific evidence and opinions for the prevention and treatment of CIAD.

## Materials and methods

### Data source and participants

NHANES is a national study investigating the nutrition and health status of Americans with a 2-year survey cycle, which is a free and open database that researchers around the world can download for free for academic research analysis. The entire research study passed the ethical review of the National Center for Health Statistics (NCHS) in the United States, and informed consent was obtained from all participants.

**Fig. 1:**
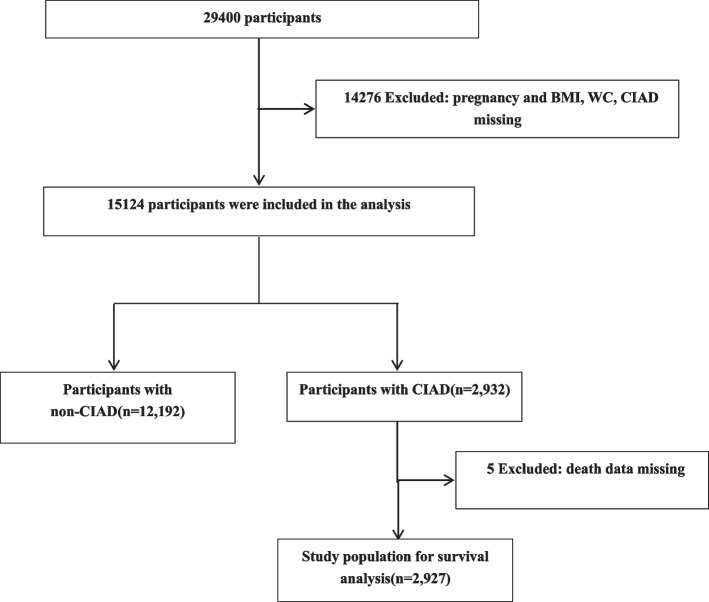
Flowchart of participants’ selection. BMI: body mass index; WC: waist circumference; CIAD: chronic inflammatory airway disease

Adults over 20 who answered the chronic inflammatory airway disease questionnaire between 2013 and 2018 were included in our study. Initially, a total of 29,400 patients were collected. Participants with missing data on BMI (*n* = 3584), waist circumference (WC)(*n* = 4825) and CIAD (*n* = 7674) and pregnancy (*n* = 167) were excluded. Finally, 15,124 eligible participants were included in the analysis. The specific inclusion and exclusion flow chart is shown in Fig. 1.

### Exposure variables

The values of BMI and WC were obtained from NHANES examination data (Body Measures section). BMI is calculated using the formula of weight (kg) divided by height (m) squared. Based on guidance from the National Heart, Lung, and Blood Institute (NHLBI) of the USA and the WHO, we divide BMI into four categories: underweight (< 18.5 kg/m^2^), normal weight (18.5–24.9 kg/m^2^), overweight (25–29.9 kg/m^2^), and obesity (≥ 30 kg/m^2^) [11, 12]. WC is an obesity indicator that measures visceral fat. Males > 102 cm and females > 88 cm are defined as obese, and other participants are defined as normal.

### Outcome variables

The outcome variables of this study are whether the participant had any self-reported CIAD, including COPD, asthma, and chronic bronchitis. These variables were obtained from NHANES questionnaire data in which participants had been told by a doctor or other health professional that they have or have had chronic bronchitis, asthma, or COPD. Participants who provided a clear answer of “yes” for any of CIAD were defined as participants having this disease.

### Mortality

To assess the impact of obesity on all-cause mortality in participants with CIAD, we conducted a prospective cohort analysis. In NHANES, study participants who had died were identified from the death certificate records from the National Death Index (NDI) in December 31st, 2019. All-cause mortality records for participants were obtained via the 2019 Linked Mortality File (LMF). Follow-up time was counted from the date of interview to the date of death or the end of the mortality period.

### Covariates

Information regarding covariates for analysis was collected from demographics, questionnaires, dietary, examination and laboratory data in NHANES. This study divided the covariates into four categories, including baseline demographic data (age, gender, BMI, WC, ethnicity, education level and poverty-income ratio), lifestyle (physical activity, energy intake, drinking status and smoking status), laboratory data (blood eosinophil counts), and comorbidities (hypertension and cardiovascular disease). And measurement details for all covariates are described on the NHANES website. Regarding the classification and definition of covariates, the study refers to other related studies [1, 13]. Education level was categorized as below high school, high school and above high school. Information on comorbidities, including hypertension and cardiovascular disease was self-reported. Cardiovascular diseases (CVD) included coronary heart disease, angina, heart failure, heart attack, and stroke.

### Statistical analysis

Continuous variables were presented as mean with standard deviation (mean ± SD) or as the median (interquartile range), while categorical variables were shown as absolute values and/or frequency (%). Missing data for covariates were filled using the imputation approach. According to the presence or absence of CIAD, all participants are divided into two groups. One-way analyses of variance (ANOVA) (normal distribution), Kruskal–Wallis test (skewed distribution), and χ^2^ test (categorical variables) were conducted to compare the baseline characteristics between the two groups.

In order to evaluate the relationship between BMI, WC, and CIAD, univariate and multivariate logistic regression analyses were conducted after adjusting for correlated covariates in the three models. Covariates were identified based on previous CIAD-related studies and clinical relevance. Model 1 was adjusted for age. Model 2 was adjusted for age, sex, race, poverty-income ratio (PIR), education level, energy intake and physical activity. Model 3 was adjusted for Model 2 plus smoking status, drink status, blood eosinophil counts, hypertension and CVD. Furthermore, all participants were divided into four groups based on BMI: < 18.5 kg/m^2^ (underweight), 18.5–24.9 kg/m^2^ (normal weight), 25–29.9 kg/m^2^ (overweight), and ≥ 30 kg/m^2^ (obesity), and normal weight was used as a reference for comparison. And according to WC, all participants were divided into obese group and normal group. To explore the possible non-linear dose–response association between BMI, WC and CIAD, we further conducted restricted cubic spline to develop smooth curves. In addition, we performed stratified analyzes among different subgroups.

In prospective cohort analysis, cox proportional-hazards regression models were employed to estimate the association between obesity and all-cause mortality. Additionally, we utilized restricted cubic spline models to generate smooth curves to assess potential nonlinear dose–response relationship between BMI, WC and all-cause mortality in patients with CIAD. In the models, BMI and WC were treated as a continuous variable with four knots (at the 5th, 35th, 65th, and 95th percentiles), as recommended by Harrell [14]. Nonlinearity was assessed using a likelihood ratio test, comparing the model with only a linear term against the model with both linear and cubic spline terms. If a nonlinear association was detected, a two-piecewise regression model was conducted to calculate the threshold effect of BMI and WC on all-cause mortality, as depicted in the smoothing plot.

All analyses were performed with R Statistical Software (https://www.R-project.org, The R Foundation) and Free Statistics software versions 1.9. A two-tailed *P* < 0.05 was considered to be significant for all analyses.

## Results

### Baseline characteristics

As shown in Fig. 1, a total of 15,124 participants were included in the final analysis, including 2932 individuals with CIAD and 12,192 non-CIAD participants. In the prospective cohort study, 5 CIAD participants with missing mortality follow-up data were excluded from survival analysis. Finally, a total of 2927 participants were included in survival analysis. The baseline characteristics of all participants were shown in Table 1. The average age of the study population was 49.8 ± 17.4 years and 7398 (48.9%) were male. The prevalence rates of CIAD, asthma, chronic bronchitis, and COPD were 19.4%, 14.9%, 5.9% and 3.7%. The mean BMI was 29.4 ± 7.0 kg/m^2^, and the mean WC was 100.0 ± 16.7 cm. Compared to the non-CIAD participants, the participants with CIAD were more likely to be older, to be female and to have higher BMI and WC. Participants in the CIAD group were more likely to smoke.

**Table 1:** Baseline characteristics of adult participants in NHANES 2013–2018

Variables	Total (*n* = 15,124)	Non CIAD(*n* = 12,192)	CIAD (*n* = 2932)	*P-value*
BMI, kg/m^2^	29.4 ± 7.0	29.0 ± 6.7	30.9 ± 8.1	< 0.001
WC, cm	100.0 ± 16.7	99.1 ± 16.2	103.6 ± 18.5	< 0.001
Age, years	49.8 ± 17.4	49.5 ± 17.3	50.6 ± 17.8	0.002
Sex, *n* (%)				< 0.001
Male	7398 (48.9)	6116 (50.2)	1282 (43.7)	
Female	7726 (51.1)	6076 (49.8)	1650 (56.3)	
Race/ethnicity, *n* (%)				< 0.001
Mexican American	2227 (14.7)	1954 (16)	273 (9.3)	
Other Hispanic	1602 (10.6)	1319 (10.8)	283 (9.7)	
Non-Hispanic White	5589 (37.0)	4236 (34.7)	1353 (46.1)	
Non-Hispanic Black	3248 (21.5)	2571 (21.1)	677 (23.1)	
Other race	2458 (16.3)	2112 (17.3)	346 (11.8)	
poverty-income ratio, *n* (%)				< 0.001
< 1.0	2823 (20.7)	2161 (19.8)	662 (24.6)	
1.0–2.99	5824 (42.7)	4648 (42.5)	1176 (43.7)	
≥ 3.0	4981 (36.5)	4126 (37.7)	855 (31.7)	
Education level, *n* (%)				0.026
Below high school	3217 (21.3)	2635 (21.6)	582 (19.9)	
High school	3428 (22.7)	2718 (22.3)	710 (24.2)	
Above high school	8467 (56.0)	6831 (56.1)	1636 (55.9)	
Energy intake, kcal/day	2110.2 ± 1003.2	2114.7 ± 993.0	2092.0 ± 1044.2	0.288
Smoking status, *n* (%)				< 0.001
Never smoker	8655 (57.3)	7326 (60.1)	1329 (45.4)	
Former smoker	2936 (19.4)	2150 (17.6)	786 (26.8)	
Current smoker	3524 (23.3)	2709 (22.2)	815 (27.8)	
Drinking status, *n* (%)				< 0.001
Never drinker	1964 (13.9)	1664 (14.7)	300 (10.8)	
Former drinker	2523 (17.9)	1932 (17)	591 (21.2)	
Mild drinker	4876 (34.5)	3941 (34.7)	935 (33.6)	
Moderate drinker	3240 (22.9)	2580 (22.7)	660 (23.7)	
Heavier drinker	1523 (10.8)	1226 (10.8)	297 (10.7)	
Physical activity, *n* (%)				< 0.001
Inactive	8651 (57.5)	7074 (58.3)	1577 (54.1)	
Insufficiently active	3793 (25.2)	2975 (24.5)	818 (28.1)	
Active	2599 (17.3)	2081 (17.2)	518 (17.8)	
hypertension, *n* (%)				< 0.001
No	10,624 (70.3)	8813 (72.4)	1811 (61.8)	
Yes	4478 (29.7)	3359 (27.6)	1119 (38.2)	
CVD, *n* (%)				< 0.001
No	13,464 (89.4)	11,107 (91.5)	2357 (80.9)	
Yes	1589 (10.6)	1033 (8.5)	556 (19.1)	
Asthma, *n* (%)				< 0.001
No	12,873 (85.1)	12,192 (100)	681 (23.2)	
Yes	2251 (14.9)	0 (0)	2251 (76.8)	
Chronic bronchitis, *n* (%)				< 0.001
No	14,230 (94.1)	12,192 (100)	2038 (69.5)	
Yes	894 (5.9)	0 (0)	894 (30.5)	
COPD, *n* (%)				< 0.001
No	14,559 (96.3)	12,192 (100)	2367 (80.7)	
Yes	565 (3.7)	0 (0)	565 (19.3)	
Blood eosinophil counts, 10^9^/L	0.2 (0.1, 0.3)	0.2 (0.1, 0.2)	0.2 (0.1, 0.3)	< 0.001

Data are presented as the mean ± SD or median (interquartile range) for skewed variables or as numbers (%) for categorical variables

*CIAD* chronic inflammatory airway disease, *BMI* body mass index, *WC* waist circumference, *CVD* Cardiovascular diseases, *COPD* chronic obstructive pulmonary disease

### Associations between BMI, WC and CIAD

**Fig. 2:**
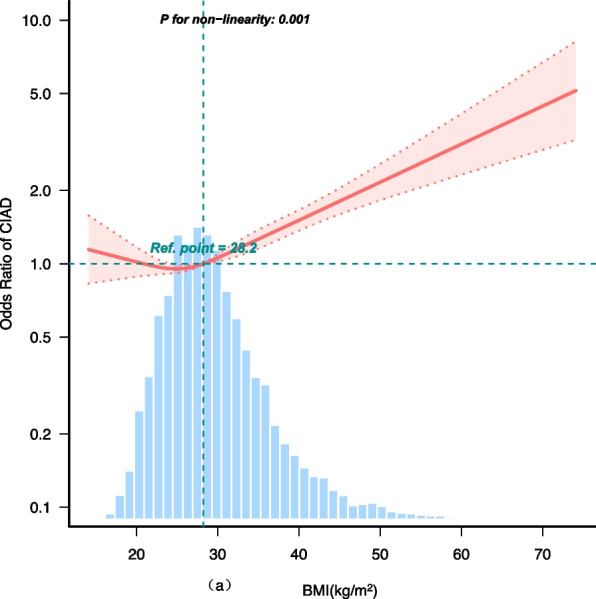
Restricted cubic spline analyses the association of BMI (**a**), WC (**b**) and the risk of chronic inflammatory airway diseases (CIAD). Adjusted for age, sex, race, PIR, education level, energy intake, physical activity, smoking status, drinking status, blood eosinophil counts, hypertension and CVD. Abbreviations: BMI: body mass index; PIR: poverty-income ratio; CVD: Cardiovascular diseases

The associations between BMI, WC and CIAD are presented in Table 2. We found that BMI and WC have a significant association with the risk of CIAD (including asthma, chronic bronchitis and COPD). After adjusting for potential covariates, it was found that for each unit increase in BMI, the incidence of CIAD increased to 2.7%. And for each unit increase in WC, the incidence of CIAD increased to 1.2%. Stratifying according to BMI, and using normal weight group as a reference, we found that the risk of CIAD in underweight and obese groups increased by 39% and 42%, respectively (p for trend < 0.001). The incidence of CIAD is not increased in overweight group (OR = 1.06, 95% CI: 0.94–1.19; *P* = 0.32). Stratifying according to WC, we found that the risk of CIAD in obese population increased by 20% (OR = 1.20, 95% CI: 1.09–1.31; *P* < 0.001). Furthermore, the dose–response relationship between BMI, WC and incidence of CIAD is presented in Fig. 2. There were non-linear associations between BMI, WC and CIAD (P for nonlinearity < 0.05).

**Table 2:** Multivariate logistic regression analyses of obesity and CIAD

	Model 1		Model 2		Model 3	
	OR (95%CI)	*P-value*	OR (95%CI)	*P-value*	OR (95%CI)	*P-value*
**BMI (kg/m**^**2**^**)**						
Continuous	1.04 (1.03–1.04)	< 0.001	1.03 (1.03–1.04)	< 0.001	1.03 (1.02–1.03)	< 0.001
Clinical cutoffs						
< 18.5	1.64 (1.20–2.25)	0.0018	1.50 (1.09–2.06)	0.0118	1.39 (1.01–1.93)	0.045
18.5–24.9	1(Ref)		1(Ref)		1(Ref)	
25–29.9	1.04 (0.93–1.17)	0.4871	1.08 (0.97–1.22)	0.1686	1.06 (0.94–1.19)	0.316
≥ 30	1.56 (1.41–1.73)	< 0.001	1.57 (1.41–1.75)	< 0.001	1.42 (1.27–1.58)	< 0.001
Trend test	1.16 (1.12–1.20)	< 0.001	1.16 (1.12–1.20)	< 0.001	1.12 (1.08–1.16)	< 0.001
**WC (cm)**						
Continuous	1.02 (1.01–1.02)	< 0.001	1.02 (1.01–1.02)	< 0.001	1.01 (1.01–1.01)	< 0.001
Clinical cutoffs						
Normal	1(Ref)		1(Ref)		1(Ref)	
Obese	1.43 (1.31–1.56)	< 0.001	1.32 (1.20–1.45)	< 0.001	1.20 (1.09–1.31)	< 0.001

Model 1 was adjusted for age; Model 2 was adjusted for age, sex, race, PIR, education level, energy intake and physical activity; Model 3 was adjusted for Model 2 plus smoking status, drink status, blood eosinophil counts, hypertension and CVD. Normal weight group is the reference group

*CIAD* chronic inflammatory airway disease, *BMI* body mass index, *WC* waist circumference, *PIR* poverty-income ratio, *CVD* Cardiovascular disease

### Subgroup analysis

**Fig. 3:**
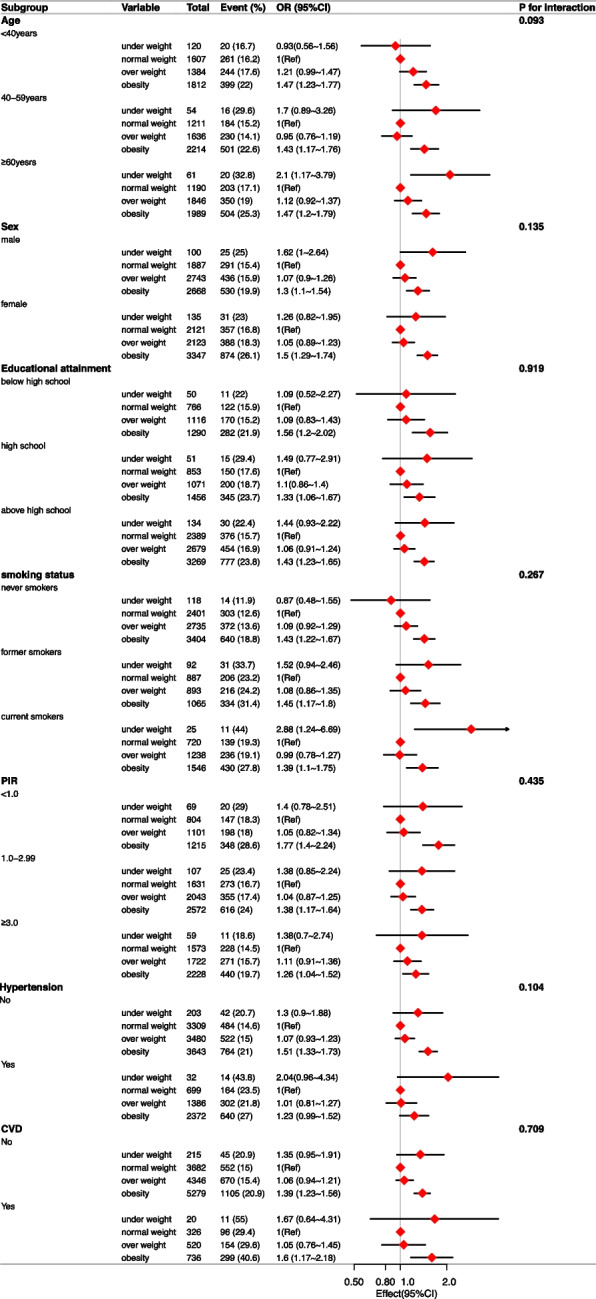
Subgroup analysis of the association between BMI and the risk of CIAD. Adjusted for age, sex, race, PIR, education level, energy intake, physical activity, smoking status, drinking status, blood eosinophil counts, hypertension and CVD. Abbreviations: BMI: body mass index; PIR: poverty-income ratio; CVD: Cardiovascular diseases

**Fig. 4:**
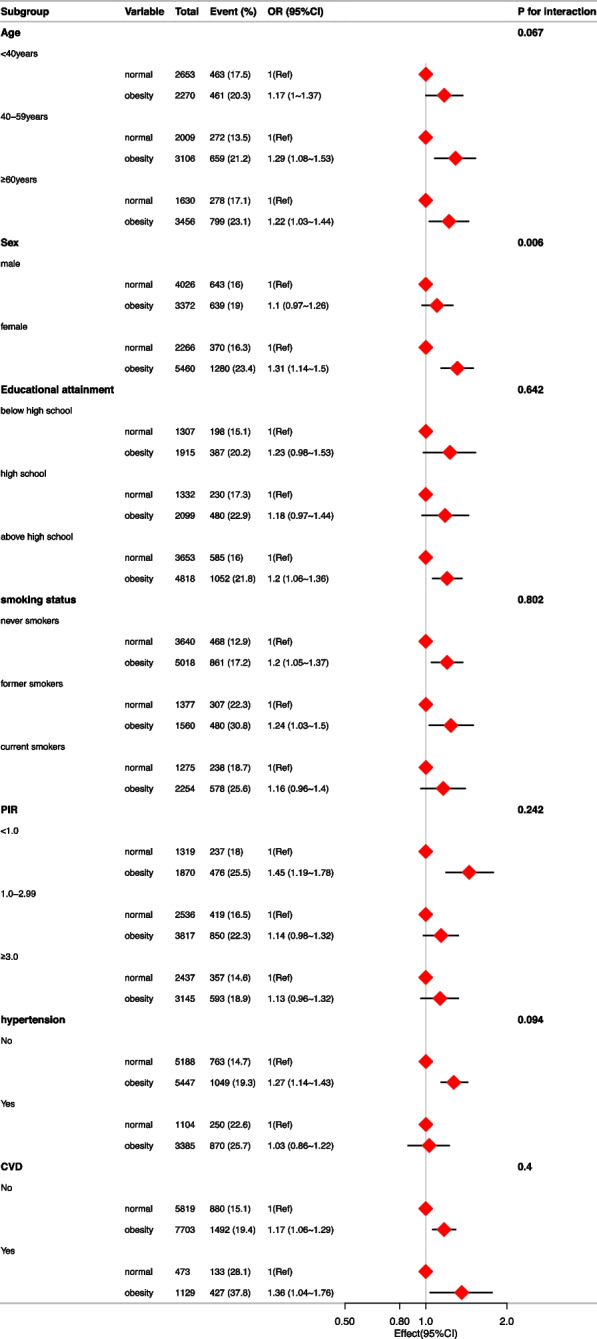
Subgroup analysis of the association between WC and the risk of CIAD. Adjusted for age, sex, race, PIR, education level, energy intake, physical activity, smoking status, drinking status, blood eosinophil counts, hypertension and CVD. Abbreviations: BMI: body mass index; PIR: poverty-income ratio; CVD: Cardiovascular diseases

In order to further assess the potential association between BMI,WC and CIAD, we conducted the subgroup analysis (Figs. 3 and 4), with stratification factors including sex (male, female), age (< 40, 40–59, ≥ 60), PIR (< 1.0, 1.0–2.99, ≥ 3.0), educational attainment (below high school, high school, above high school), physical activity (inactive, insufficiently active, active), smoking status (never smokers, former smokers, current smokers), hypertension (yes, no), CVD (yes, no). In the stratified analysis of BMI, all subgroups had no significant interactions. The result revealed that the relationship between obesity and CIAD is consistent in all subgroups (all *P* for interactions > 0.05). In the stratified analysis of WC, the result found that there was a significant association between obesity with CIAD in female population (OR = 1.31, 95%CI:1.14–1.5, *P* < 0.001) but not in male population (OR = 1.1, 95%CI:0.97–1.26, *P* = 0.15) (*P* for interactions < 0.05). There were no interactions in other subgroups.

### Associations between obesity and mortality among adults with CIAD

In prospective cohort study, the median follow-up time is 47 months (mean ± SD, 48.1 ± 21.1 months). There are 186 (6.4%) all-cause deaths among 2,927 participants with CIAD. After cox regression analysis, we found that compared with normal weight group, underweight was associated with a higher mortality (HR = 2.44, 95% CI:1.28–4.44, *P* = 0.005), whereas overweight (HR = 0.58, 95% CI:0.39–0.87, *P* = 0.009) and obesity (HR = 0.59, 95% CI:0.4–0.87, *P* = 0.008) were associated with a lower mortality (*P* for trend < 0.05). And the results were the same when stratified by waist circumference (HR = 0.68, 95% CI = 0.49–0.95, *P* = 0.023). The results are presented in Table 3.

**Table 3:** Multivariable-adjusted HRs and 95% CIs for obesity in relation to all-cause mortality

	Model 1		Model 2		Model 3	
	HR (95%CI)	*P-value*	HR (95%CI)	*P-value*	HR (95%CI)	*P-value*
**BMI (kg/m**^**2**^**)**						
< 18.5	3.1 (1.7–5.62)	< 0.001	2.85 (1.56–5.22)	0.001	2.44 (1.31–4.55)	0.005
18.5–24.9	1(Ref)		1(Ref)		1(Ref)	
25–29.9	0.66 (0.45–0.98)	0.04	0.65 (0.43–0.96)	0.032	0.58 (0.39–0.87)	0.009
≥ 30	0.71 (0.49–1.02)	0.066	0.7 (0.48–1.02)	0.065	0.59 (0.4–0.87)	0.008
Trend test	0.86 (0.76–0.97)	0.012	0.86 (0.76–0.97)	0.013	0.82 (0.72–0.93)	0.002
**WC (cm)**						
Normal	1(Ref)		1(Ref)		1(Ref)	
Obese	0.7 (0.52–0.94)	0.02	0.77 (0.56–1.06)	0.107	0.68 (0.49–0.95)	0.023

Model 1 was adjusted for age; Model 2 was adjusted for age, sex, race, PIR, education level, energy intake and physical activity; Model 3 was adjusted for Model 2 plus smoking status, drinking status, blood eosinophil counts, hypertension and CVD

*Abbreviations*: Normal weight group is the reference group. *BMI* body mass index, *WC* waist circumference, *PIR* poverty-income ratio, *CVD* Cardiovascular diseases

**Fig. 5:**
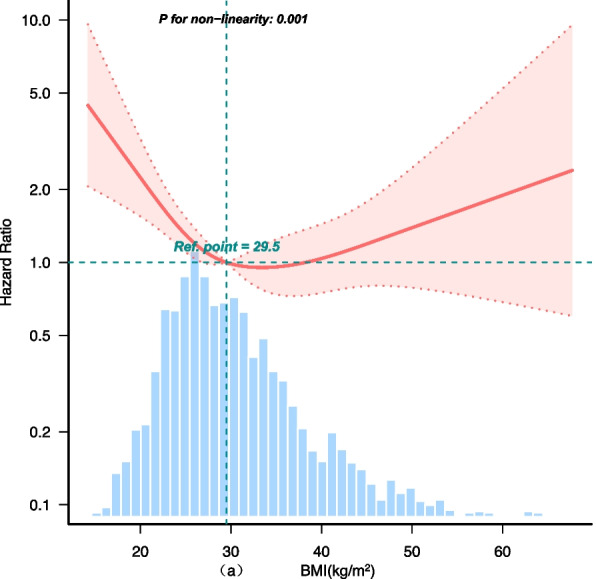
Restricted cubic spline analyses the association of BMI (**a**), WC (**b**) and all-cause mortality in patients with chronic inflammatory airway diseases (CIAD). Adjusted for age, sex, race, PIR, education level, energy intake, physical activity, smoking status, drinking status, blood eosinophil counts, hypertension and CVD. Abbreviations: BMI: body mass index; PIR: poverty-income ratio; CVD: Cardiovascular diseases

By restricted cubic spline model and smooth curve fitting (after adjusting for age, gender, race, PIR, education level, energy intake, physical activity, smoking status, drinking status, blood eosinophil counts, hypertension and CVD), we found that there was a non-linear association between BMI and all-cause mortality (*P* for non-linear = 0.001, Fig. 5). A turning point value of BMI (32.4 kg/m^2^) was found by a segmentation regression model between BMI and all-cause mortality. The mortality of CIAD patients was lowest when BMI was 32.4 kg/m^2^. When BMI ≤ 32.4 kg/m^2^, BMI was inversely associated with all-cause mortality in patients with CIAD (HR: 0.92, 95%CI:0.88–0.97, *P* < 0.001). However, when BMI > 32.4 kg/m^2^, there was no association between BMI and all-cause mortality (HR:1.02, 95%CI:0.97–1.06, *P* = 0.52). *P* value for likelihood ratio test (LRT) was 0.001. The results indicated that model II (two-segment non-linear model) was more suitable to describe the relationship between BMI and the risk of all-cause mortality in CIAD patients, as presented in Table 4.

**Table 4:** Threshold effect analysis for the relationship between BMI and all-cause mortality

	BMI (kg/m^2^) groups	All-cause mortality	
		HR (95%CI)	*P-value*
Model I: The linear model	< 18.5	2.44 (1.31–4.55)	0.005
	18.5–24.9	1(Ref)	
	25–29.9	0.58 (0.39–0.87)	0.009
	≥ 30	0.59 (0.4–0.87)	0.008
	Trend test	0.82 (0.72–0.93)	0.002
Model II: Two-segment non-linear model	The turning point of BMI (kg/m^2^)		
	≤ 32.4 (slope1)	0.92 (0.88–0.97)	< 0.001
	> 32.4 (slope2)	1.02 (0.97–1.06)	0.52
	LRT		0.001

Adjusted for age, sex, race, PIR, education level, energy intake, physical activity, smoking status, drinking status, blood eosinophil counts, hypertension and CVD. Normal weight group is the reference group

*Abbreviations*: *BMI* body mass index, *PIR* poverty-income ratio, *CVD* Cardiovascular diseases, *LRT* likelihood ratio test

## Discussion

We conducted a cross-sectional study to analyze the relationship between obesity and the incidence of CIAD using NHANES 2013–2018 data. The results found that a positive association between BMI, WC and the risk of CIAD, which were non-linear associations. When grouped by BMI and WC, we found that underweight and obesity were positively associated with a higher prevalence of CIAD compared with normal weight. Grouped according to BMI, this positive association between obesity and the risk of CIAD remain robust after adjusting for related variables and consistent across all subgroups. Grouped according to WC, a significant association between obesity and the risk of CIAD was revealed only in female population, not in male population.

Several studies confirmed that obesity is an independent risk factor for chronic respiratory diseases, especially asthma and COPD [15–18]. A Mendelian randomization study revealed that obesity increases the possibility of COPD (BMI: OR = 1.429; WC: OR = 1.591), asthma (BMI: OR = 1.358; WC: OR = 1.515) and acute bronchitis (BMI: OR = 1.252;WC: OR = 1.237) [19]. A cross-sectional survey has demonstrated that obesity is related to symptoms of dyspnea, and about 1/4 of dyspnea symptoms are caused by obesity [20]. This is consistent with the research results of Marta et al. [16], who found that both general and abdominal obesity were associated with respiratory symptoms. Additionally, they divided obese people into abdominal obesity and general obesity based on WC and BMI. The result revealed that after adjusting for relevant covariates, abdominal obesity was independently associated with asthma and chronic bronchitis, while general obesity was significantly associated with COPD and asthma after adjustment for abdominal obesity. Both a cohort study and a prospective study have affirmed that in the asthmatic population, individuals with obesity have lower predictive values of forced expiratory volume in one second (FEV1) and forced vital capacity (FVC) compared with individuals with non-obesity [21, 22]. Moreover, obesity increases frequency and severity of asthma exacerbations [23]. However, a cross-sectional study from western China suggested that overweight and obesity are protective factors for COPD, the odds ratio (OR) were 0.614 (95% CI 0.517–0.730, *P* < 0.001) and 0.572 (95% CI 0.449–0.721, *P* < 0.001), respectively. The inconsistent results may be due to inconsistencies in study populations and sample sizes. Therefore, we need more studies with larger sample sizes and more rigorous research designs.

Obesity is the consequence of an excessive accumulation of adipose tissue, which is an active endocrine organ that can secrete a variety of cytokines and hormones [24–26]. CIAD (including COPD, asthma and chronic bronchitis) are closely related to inflammation, while adipokine that promotes the occurrence and development of inflammation mainly originates from adipose tissue. Therefore, as a low-grade systemic inflammation driven by phenotypic changes in adipose tissue-related macrophages, obesity is very likely to cause inflammatory airway diseases such as asthma and COPD [27, 28]. On the other hand, patients with obesity have an increase in peripheral blood leukocytes, which may lead to increased inflammation and the production of more pro-inflammatory mediators [29]. The inflammatory process underlying both diseases could be one of the potential connecting links between both diseases. However, the precise relevance of inflammation as a mediator between both processes has yet to be fully elucidated [30]. Additionally, obesity has an impact on the ventilatory mechanics of the population, which may play an important role in the association between CIAD and obesity [31].

In our prospective cohort study, results suggest that BMI and WC are inversely related to mortality in population with CIAD. Moreover, the CIAD mortality rate among underweight population was nearly 2.5 times higher than that among normal-weight, while overweight and obesity were significantly associated with a decreased risk of all-cause mortality compared to normal weight population.

Obesity is a major risk factor for chronic non-communicable diseases worldwide and is associated with increased diseases incidence and mortality [7, 23, 32]. Obesity can lead to low-grade chronic systemic inflammation, which predisposes individuals to an increased risk of morbidity and mortality. In contrast, numerous researches have shown that obesity is a protective factor for COPD mortality [10, 33], which is known as the "obesity paradox". Yet the relationships between obesity and mortality in patients with COPD and asthma were also inconsistent [34]. A dose–response meta-analysis revealed a nonlinear dose–response relationship between BMI and mortality in COPD patients [35]. When the BMI was 30 kg/m^2^, the mortality rate was lowest, and when the BMI was greater than 32 kg/m^2^, the BMI was not related to the mortality rate. The results are basically consistent with our study. We found there was a non-linear association between BMI and all-cause mortality, and the turning point value of BMI was 32.4 kg/m^2^. The mortality of CIAD patients was lowest when BMI was 32.4 kg/m^2^. When BMI ≤ 32.4 kg/m^2^, all-cause mortality decreased with increasing BMI. However, when BMI > 32.4 kg/m^2^, there was no association between BMI and all-cause mortality.

There are many different explanations for the obesity paradox, but there is currently no unified understanding. One of the most accepted explanations is that current obesity indicators cannot effectively distinguish body composition, including fat mass and muscle mass [10, 36]. Another explanation is the influence of confounding factors, such as smoking status. Research has found that the obesity paradox does not exist in non-smoking patients [37]. At last, most researches ignore the possible bias caused by obesity levels. Our study suggests that when BMI ≤ 32.4 kg/m^2^, obesity was positively associated with all-cause mortality, while when BMI > 32.4 kg/m^2^, obesity has no significant association with all-cause mortality.

The major strength of our study is that we used both BMI and waist circumference to analysis the relationship between obesity and CIAD. BMI and waist circumference are predictors of different obesity types, which can avoid some possible biases. Second, our study is representative of a large sample of the population, which made our research conclusions more reliable. Third, in this study, we established three models, adjusting for major covariates that may affect the risk of CIAD, including age, gender, ethnicity, poverty-income ratio, education level, physical activity, energy intake, drinking status, smoking status, blood eosinophil counts, and comorbidities (hypertension and cardiovascular disease). After adjusting for potential confounders, our findings remained relatively stable. At last, we collected death data on CIAD patients to analysis the association between different body weights and the risk of all-cause mortality. Additionally, our conducted two models (the linear model and two-segment non-linear model) to analyze the association between BMI and all-cause mortality and found the turning point of BMI. This may contribute to providing an appropriate weight range for the prevention and prognosis of CIAD.

There are several limitations in this study. First, this study is an observational study and cannot determine the causal relationship between obesity and CIAD. Second, the diagnosis of CIAD came from self-reported questionnaires, which is prone to bias and may miss some patients who have never seen a doctor. Third, in multivariate analysis and cox regression analysis, we adjusted for many major confounders, but there may still be some unknown confounders. Moreover, we adjusted for smoking status among all participants, but we did not conduct stratified analyzes by smoking amount and smoking duration, which may cause certain biases. Finally, we investigated the association between obesity and the incidence of total CIAD (including asthma, COPD and chronic bronchitis) as well as all-cause mortality in patients with CIAD. CIAD (including asthma, COPD and chronic bronchitis) is not a specific respiratory disease, but a type of respiratory disease, mainly related to airway inflammatory response. There have been numerous researchers on the link between a particular respiratory disease and obesity. However, the aim of this study is to investigate the relationship between obesity and a certain type of respiratory disease to provide an overall impression and to facilitate further research.

## Conclusion

In conclusion, our study found that underweight and obesity were associated with the increased risk of CIAD compared to normal weight. Underweight was associated with increased all-cause mortality, while overweight was associated with reduced all-cause mortality. There was a non-linear association between BMI and all-cause mortality in patients with CIAD. The mortality of CIAD patients was lowest when BMI was 32.4 kg/m^2^. When BMI ≤ 32.4 kg/m^2^, BMI was positively associated with all-cause mortality, while when BMI > 32.4 kg/m^2^, BMI had no significant association with all-cause mortality. Therefore, overweight and mild obesity (BMI ≤ 32.4 kg/m^2^) are beneficial for the prognosis of CIAD, while being underweight is detrimental to the prognosis of CIAD.

## Data Availability

Publicly available datasets were analyzed in this study. This data can be found here: https://www.cdc.gov/nchs/nhanes/search/default.aspx.

## References

[1] Lin S, Zhu N, Zhang S (2023). Associations of dietary fiber intake with chronic inflammatory airway diseases and mortality in adults: a population-based study. Front Public Health.

[2] GBD Chronic Respiratory Disease Collaborators (2020). Prevalence and attributable health burden of chronic respiratory diseases, 1990–2017: a systematic analysis for the Global Burden of Disease Study 2017. Lancet Respir Med.

[3] Li X, Cao X, Guo M, Xie M, Liu X (2020). Trends and risk factors of mortality and disability adjusted life years for chronic respiratory diseases from 1990 to 2017: systematic analysis for the Global Burden of Disease Study 2017. BMJ.

[4] Safiri S, Carson-Chahhoud K, Noori M, Nejadghaderi SA, Sullman MJM, Ahmadian Heris J (2022). Burden of chronic obstructive pulmonary disease and its attributable risk factors in 204 countries and territories, 1990–2019: results from the Global Burden of Disease Study 2019. BMJ.

[5] Stern J, Pier J, Litonjua AA (2020). Asthma epidemiology and risk factors. Semin Immunopathol.

[6] Chong B, Jayabaskaran J, Kong G, Chan YH, Chin YH, Goh R (2023). Trends and predictions of malnutrition and obesity in 204 countries and territories: an analysis of the Global Burden of Disease Study 2019. EClinicalMedicine.

[7] Obesity n.d. https://www.who.int/health-topics/obesity. Accessed 20 Jan 2024.

[8] Al Hennawi H, Zohaib M, Khan MK, Ahmed F, Mathbout OF, Alkhachem A (2024). Temporal trends in obesity-related mortality rates: an analysis of gender, race/ethnicity, and geographic disparities in the United States. Curr Probl Cardiol.

[9] Zhang X, Chen H, Gu K, Jiang X (2022). Association of body mass index and abdominal obesity with the risk of airflow obstruction: National Health and Nutrition Examination Survey (NHANES) 2007–2012. COPD.

[10] Brock JM, Billeter A, Müller-Stich BP, Herth F (2020). Obesity and the lung: what we know today. Respir Int Rev Thorac Dis.

[11] Clinical Guidelines on the Identification, Evaluation, and Treatment of Overweight and Obesity in Adults- -The Evidence Report (1998). National institutes of health. Obes Res.

[12] WHO Consultation on Obesity. Obesity: preventing and managing the global epidemic. Report of a WHO consultation. World Health Organ Tech Rep Ser. 2000;894:i-xii, 1–253. 11234459

[13] Zhao D, Chen P, Chen M, Chen L, Wang L (2024). Association of magnesium depletion score with congestive heart failure: results from the NHANES 2007–2016. Biol Trace Elem Res.

[14] Harrell FE (2015). Regression modeling strategies: with applications to linear models, logistic regression, and survival analysis.

[15] Martin-Rodriguez E, Guillen-Grima F, Martí A, Brugos-Larumbe A (2015). Comorbidity associated with obesity in a large population: The APNA study. Obes Res Clin Pract.

[16] Kisiel MA, Arnfelt O, Lindberg E, Jogi O, Malinovschi A, Johannessen A (2023). Association between abdominal and general obesity and respiratory symptoms, asthma and COPD. Results from the RHINE study. Respir Med.

[17] Ramos-Nino ME, MacLean CD, Littenberg B (2021). Association between prevalence of obstructive lung disease and obesity: results from The Vermont Diabetes Information System. Asthma Res Pract.

[18] Baniya S, Shrestha TM, Pant P, Aacharya RP (2023). Metabolic syndrome among stable chronic obstructive pulmonary disease patients visiting outpatient department of a tertiary care centre: A descriptive cross-sectional study. JNMA J Nepal Med Assoc.

[19] Yang W, Yang Y, Guo Y, Guo J, Ma M, Han B (2023). Obesity and risk for respiratory diseases: a Mendelian randomization study. Front Endocrinol.

[20] Guo YL, Ampon MR, Poulos LM, Davis SR, Toelle BG, Marks GB (2023). Contribution of obesity to breathlessness in a large nationally representative sample of Australian adults. Respirol Carlton Vic.

[21] Kasteleyn MJ, Bonten TN, de Mutsert R, Thijs W, Hiemstra PS, le Cessie S (2017). Pulmonary function, exhaled nitric oxide and symptoms in asthma patients with obesity: a cross-sectional study. Respir Res.

[22] Alqarni AA, Aldhahir AM, Siraj RA, Alqahtani JS, Alshehri HH, Alshamrani AM (2023). Prevalence of overweight and obesity and their impact on spirometry parameters in patients with asthma: A multicentre, retrospective study. J Clin Med.

[23] Tashiro H, Kurihara Y, Kuwahara Y, Takahashi K (2024). Impact of obesity in asthma: Possible future therapies. Allergol Int Off J Jpn Soc Allergol.

[24] Ping Z, Pei X, Xia P, Chen Y, Guo R, Hu C (2018). Anthropometric indices as surrogates for estimating abdominal visceral and subcutaneous adipose tissue: A meta-analysis with 16,129 participants. Diabetes Res Clin Pract.

[25] Sideleva O, Suratt BT, Black KE, Tharp WG, Pratley RE, Forgione P (2012). Obesity and asthma: an inflammatory disease of adipose tissue not the airway. Am J Respir Crit Care Med.

[26] Lumeng CN, Saltiel AR (2011). Inflammatory links between obesity and metabolic disease. J Clin Invest.

[27] Martinez-Santibañez G, Lumeng CN-K (2014). Macrophages and the regulation of adipose tissue remodeling. Annu Rev Nutr.

[28] Woo J, Koziol-White C, Panettieri R, Jude J (2021). TGF-β: The missing link in obesity-associated airway diseases?. Curr Res Pharmacol Drug Discov.

[29] Mancuso P (1985). Obesity and lung inflammation. J Appl Physiol Bethesda Md.

[30] Bantulà M, Roca-Ferrer J, Arismendi E, Picado C (2021). Asthma and obesity: two diseases on the rise and bridged by inflammation. J Clin Med.

[31] Grassi L, Kacmarek R, Berra L (2020). Ventilatory mechanics in the patient with obesity. Anesthesiology.

[32] Dai H, Alsalhe TA, Chalghaf N, Riccò M, Bragazzi NL, Wu J (2020). The global burden of disease attributable to high body mass index in 195 countries and territories, 1990–2017: An analysis of the Global Burden of Disease Study. PLoS Med.

[33] Cao C, Wang R, Wang J, Bunjhoo H, Xu Y, Xiong W (2012). Body mass index and mortality in chronic obstructive pulmonary disease: a meta-analysis. PLoS One.

[34] Yano C, Kawayama T, Kinoshita T, Tokunaga Y, Sasaki J, Sakazaki Y (2021). Overweight improves long-term survival in Japanese patients with asthma. Allergol Int Off J Jpn Soc Allergol.

[35] Guo Y, Zhang T, Wang Z, Yu F, Xu Q, Guo W (2016). Body mass index and mortality in chronic obstructive pulmonary disease: A dose-response meta-analysis. Medicine (Baltimore).

[36] Chan SMH, Selemidis S, Bozinovski S, Vlahos R (2019). Pathobiological mechanisms underlying metabolic syndrome (MetS) in chronic obstructive pulmonary disease (COPD): clinical significance and therapeutic strategies. Pharmacol Ther.

[37] Wu TD, Ejike CO, Wise RA, McCormack MC, Brigham EP (2019). Investigation of the obesity paradox in chronic obstructive pulmonary disease, according to smoking status, in the United States. Am J Epidemiol.

